# Identification of hsa-miR-335 as a Prognostic Signature in Gastric Cancer

**DOI:** 10.1371/journal.pone.0040037

**Published:** 2012-07-03

**Authors:** Zhi Yan, Yimin Xiong, Weitian Xu, Juan Gao, Yi Cheng, Zhigang Wang, Fang Chen, Guorong Zheng

**Affiliations:** 1 Department of Digestive Diseases, Wuhan General Hospital of Guangzhou Command, Wuhan, Hubei, China; 2 Department of Oncology, Wuhan General Hospital of Guangzhou Command, Wuhan, Hubei, China; 3 Department of Medical Laboratory, Wuhan General Hospital of Guangzhou Command, Wuhan, Hubei, China; University of Barcelona, Spain

## Abstract

**Background:**

Gastric cancer (GC) is one of the most common malignancy and primary cause of death in Chinese cancer patients. Recurrence is a major factor leading to treatment failure and low level of 5-year survival rate in GC patients following surgical resection. Therefore, identification of biomarkers with potential in predicting recurrence risk is the key problem of the prognosis in GC patients.

**Patients and Methods:**

A total of 74 GC patients were selected for systematic analysis, consisting of 31 patients with recurrence and 43 patients without recurrence. Firstly, miRNAs microarray and bioinformatics methods were used to characterize differential expressed miRNAs from primary tumor samples. Following, we used a ROC method to select signature with best sensitivity and specificity. Finally, we validated the signature in GC samples (frozen fresh and blood samples) using quantitative PCR.

**Results:**

We have identified 12 differential miRNAs including 7 up-regulated and 5 down-regulated miRNAs in recurrence group. Using ROC method, we further ascertained hsa-miR-335 as a signature to recognize recurrence and non-recurrence cases in the training samples. Moreover, we validated this signature using quantitative PCR method in 64 test samples with consistent result with training set. A high frequency recurrence and poor survival were observed in GC cases with high level of hsa-miR-335 (*P*<0.001). In addition, we evaluated that hsa-miR-335 were involved in regulating target genes in several oncogenic signal-pathways, such as p53, MAPK, TGF-β, Wnt, ERbB, mTOR, Toll-like receptor and focal adhesion.

**Conclusion:**

Our results indicate that the hsa-miR-335 has the potential to recognize the recurrence risk and relate to the prognosis of GC patients.

## Introduction

Currently, surgery is still the main option for treating gastric cancer (GC), however even after performing curative resection, approximately 40–65% of GC patients will experience a recurrence of the disease possibly encompassing local relapse, peritoneal dissemination or hematogenous metastasis [1], [2]. For this reason, the 5-year survival rate of GC patients was still in a low level. A major challenge for improving clinical outcomes is to better understand molecular mechanisms and recognize robust signatures for the prognosis of GC.

Several risk factors, including pathological serosa state, margin status, tumor microvessel density and gene expression changes related with recurrence have been reported [3], [4]. In addition, a few predictive or diagnostic methods have been used to evaluate the recurrence risk based on gene expression profiling or a set of clinical variables [5]–[8]. However, these multiple observations including several gene or protein alterations may hamper their clinical application. Reliable and convenient molecular markers for clinical practitioners are required for the prognosis in GC.

A recently discovered of miRNAs are highly conserved in the genomes of invertebrates, vertebrates and plants. They serve critical functions in multiple biological processes [9] including developmental timing, patterning and embryogenesis, differentiation, organogenesis, and apoptosis [10]. Given its multiple essential biological functions, it is not surprising that the abnormal miRNAs expression will lead to disease and even cancer [11].

miRNAs have been presented as effective potential biomarkers for tracing the tissue origin in malignant tumors of unknown primary origin [12]; miRNAs expression profiles are able to reflect the developmental lineage and differentiated state of the tumors [11]. Systematic analysis on the stability of miRNAs as well as optimized conditions for expression analysis using formalin-fixed paraffin-embedded and blood samples have been reported previously [13]–[15], shedding light on their unanticipated diagnostic potential. There are a limited number of miRNAs [16] in the human genome that are known to regulate approximately 30% of genes [17]. These attributes of miRNAs may provide us with a simple method to predict the recurrence risk for cancer patients.

In this study, we have identified a miRNA (hsa-miR-335) having potential to predict recurrence risk of GC based on microarray and clinical data of a small-size sample in Chinese GC patients. Our results showed that hsa-miR-335 could be act as a clinical prognostic signature for GC.

**Table 1:** Details of patients and tumors used in this study.

Characteristic	Recurrence group (n = 31)	Non-recurrence group (n = 43)	*P* value
Sex			
Male	22	27	
Female	9	16	*P* = 0.469
Age (year)			
Median	64	58	*P* = 0.502
Range	30–83	33–79	
Tumor location			
Cardia	9	8	*P* = 0.299
Body	7	10	
Pyloric antrum	15	25	
Differentiation			
Poor	19	23	*P* = 0.511
Moderate-poor	6	14	
Others	6	6	
Lymph node resection			
<12	25	39	*P* = 0.217
>12	6	4	
UICC stage			
I	2	8	*P* = 0.026
II	4	11	
III	20	23	
IV(M0)	5	1	
Embolus			
With	11	9	*P* = 0.169
Without	20	34	
Adjuvant chemotherapy			
Performed	12	23	*P* = 0.214
Not performed	19	20	
Patients’ status			
Survival	8	34	*P*<0.001
Death	23	9	
Median survival time (month)	18.9	43.9	*P*<0.001

## Methods

### Clinical Samples

This study has been approved by the Ethics Committee of Wuhan General Hospital of Guangzhou Command, PLA. All gastric cancer patients and non-cancer patients with digestive diseases provided written informed consent in our study.

Patients undergoing gastrectomy for potentially curable GC at the Wuhan general hospital of Guangzhou military command were subjects in this study. Eligibility for inclusion in this study included histologically confirmed adenocarcinoma of the stomach or gastroesophageal junction. All patients had received complete resections including an attempt at complete tumor removal with inclusion of wide negative margins and extended retroperitoneal lymphadenectomy (D2 type). Information on clinicopathologic, therapeutic, and outcome parameters of patients from May 2005 to Feb 2012 was collected retrospectively. Cancer staging was performed according to the fifth edition of the American Joint Commission on Cancer TNM criteria.

**Table 2:** Summary of miRNAs differential expressed in GC with/without recurrence.

Up-regulated	Fold change	Q value	Down-regulated	Fold Change	Q value
hsa-miR-335	4.675	0	hsa-miR-373	0.0969	0
hsa-miR-133a	2.115	0	hsa-miR-19a	0.1809	0
hsa-miR-133b	2.491	0	hsa-miR-142-5p	0.1462	0
hsa-miR-128b	1.453	0	hsa-miR-142-3p	0.1857	0
hsa-miR-429	2.661	0	hsa-miR-32	0.1984	0
hsa-miR-194	1.885	0			
hsa-miR-375	2.314	0			

**Figure 1:**
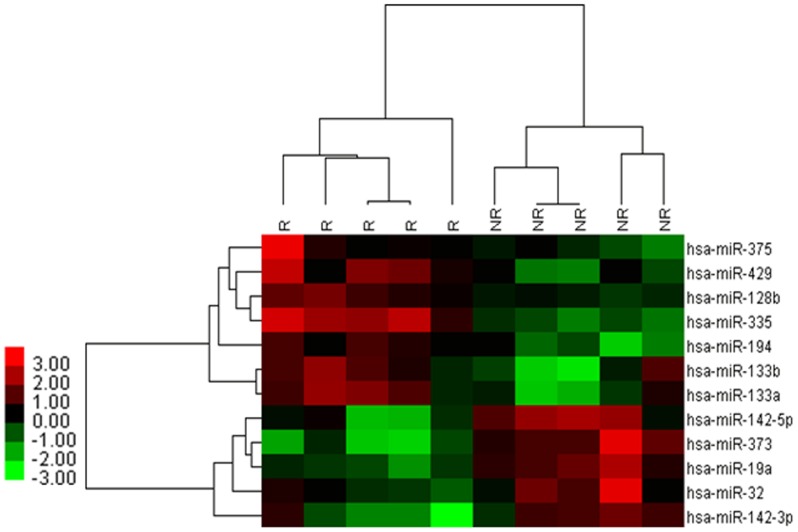
Cluster analysis of expressed miRNAs in GC samples with and without recurrence. A total of 12 differential expressed miRNAs, including 7 up-regulated and 5 down-regulated, were identified by SAM in GC samples with and without recurrence according to the criteria of FC >2, q = 0. Columns represent samples and rows represent miRNAs (black, green, and red correspond to unchanged, down-regulated and up-regulated, respectively). R: recurrence samples; NR: non-recurrence samples.

Recurrence was defined as any cancer recurrence including lymph node, remnant stomach, local, peritoneal and hematogenous metastases. All patients that experienced recurrence of cancer were diagnosed clinically or radiographically, and confirmed by biopsy via upper gastrointestinal endoscope or percutaneous puncture. The radiographic standard for the recurrence diagnosis included CT or MRI of the chest, abdomen, pelvis, head and bone scans, or other diagnostic tests which were used only under special circumstances. All of the samples were obtained from surgical specimens of patients with gastric adenocarcinoma and all patients gave written consent for the use of these tissues for research purposes. We selected samples (including frozen fresh and blood samples) from 74 patients with and without GC recurrence.

### miRNA Microarray

The miRNA microarray analysis was performed as described in detail on the website of CapitalBio (http://www.capitalbio.com) and also according to Hua’s procedure [18]. Briefly, 50–100 µg of total RNA was used to extract miRNAs using a miRNA isolation kit AM1560 (Ambion, USA). Fluorescein-labeled miRNAs were used for hybridization on Affymetrix miRNA Chip containing 1075 probes. The fluorescence signal were scanned by GeneChip Scanner 3000 7G (Affymetrix, USA). Raw data were normalized and analyzed in GeneChip Command Console 1.1 software (Affymetrix, USA).

**Figure 2:**
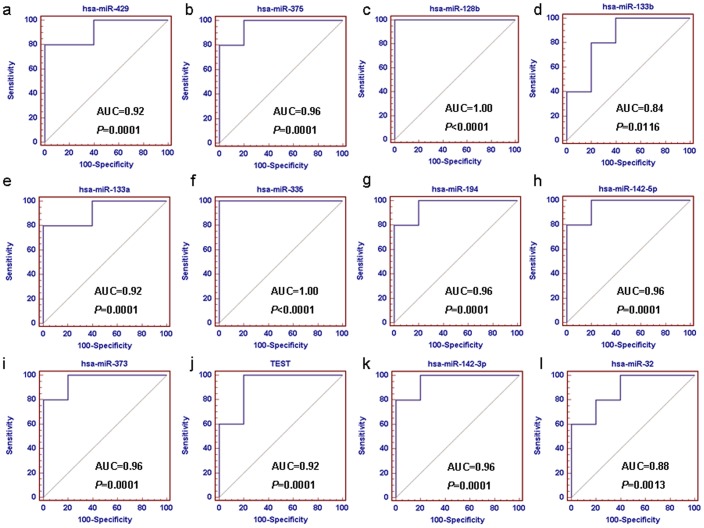
ROC analyses of the candidate biomarkers based on miRNA microarray data. We obtained 2 miRNAs hsa-miR-335 and hsa-miR-128b with best sensitivity and specificity in classifying GC samples with and without recurrence. Figures (a) to (l) represent the ROC curves of hsa-miR-429, hsa-miR-3755, hsa-miR-128b, hsa-miR-133b, hsa-miR-133a, hsa-miR-335, hsa-miR-194, hsa-miR-142-5p, hsa-miR-373, hsa-miR-19a, hsa-miR-142-3p and hsa-miR-32, respectively.

**Table 3:** ROC curves analyses of the miRNAs selected SAM.

Sample sets	miRNAs	Sensitivity	Specificity	AUC	95% CI	*P* value
Training sets	hsa-miR-429	100.0%	80.0%	0.92	0.579–0.981	0.0001
	hsa-miR-375	100.0%	80.0%	0.96	0.631–0.960	0.0001
	hsa-miR-128b	100.0%	100.0%	1.00	0.690–1.000	0
	hsa-miR-133b	80.0%	80.0%	0.84	0.486–0.979	0.0116
	hsa-miR-133a	80.0%	100.0%	0.92	0.579–0.981	0.0001
	hsa-miR-335	100.0%	100.0%	1.00	0.690–1.000	0
	hsa-miR-194	100.0%	80.0%	0.96	0.631–0.960	0.0001
	hsa-miR-142-5p	80.0%	100.0%	0.96	0.631–0.960	0.0001
	hsa-miR-373	80.0%	100.0%	0.96	0.631–0.960	0.0001
	hsa-miR-19a	100.0%	80.0%	0.92	0.579–0.981	0.0001
	hsa-miR-142-3p	80.0%	100.0%	0.96	0.631–0.960	0.0001
	hsa-miR-32	60.0%	100.0%	0.88	0.531–0.984	0.0013
Test sets	hsa-miR-335	87.1%	79.1%	0.88	0.780–0.942	0.0001
	hsa-miR-128b	80.6%	67.4%	0.79	0.677–0.874	0.0001
	hsa-miR-335/128b	87.1%	74.4%	0.84	0.739–0.916	0.0001

### RNA Extraction from Frozen Fresh and Blood Samples

RNA was extracted from frozen fresh GC tissues using a standard Trizol protocol (Invitrogen, Carlsbad, CA, USA). For blood samples, miRNA were extracted using the miRNease kit (Qiagen). In brief, 700 µl of QIAzol reagent was added to 200 µl of plasma sample and incubated for 5 min at room temperature. Then, 140 µl of chloroform was added, vortexed for 15 sec and incubated for 3 min at room temperature. This was followed by a centrifugation of 14000 rpm at 4°C for 15 min. The upper, watery phase was removed and 1.5 times of its volume added in 100% ethanol. Each 700 µl of mixture were placed on a column and centrifuged at 13000 rpm at room temperature for 15 sec. Following, 500 µl Buffer RPL was added to the column and centrifuged at 13000 rpm at room temperature for 2 min. The column was then centrifuged at 13000 rpm at room temperature to dry it. The following was the elution step with 20 µl of nuclease-free water. The eluted RNA was stored at −70°C.

**Figure 3:**
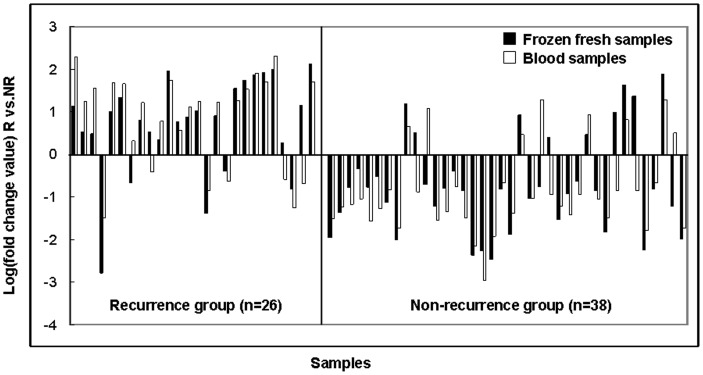
Validation of the signature in GC samples by real-time PCR. The results in frozen fresh samples showed that in 26 cases with recurrence, 21 cases with high-expressed of hsa-miR-335; and in 38 cases without recurrence, 29 cases with low-expressed of hsa-miR-335. The same results were observed in blood samples, in 26 cases with recurrence, 19 cases with high-expressed of hsa-miR-335; and in 38 cases without recurrence, 30 cases with low-expressed of hsa-miR-335. There were 11 cases were not matched in the expression level of hsa-miR-335 in comparison of the results between frozen fresh and blood samples.

### Real-time Quantitative PCR

Mature miRNA sequences were acquired from the Sanger Institute miRBase Sequence Database (http://microrna.sanger.ac.uk/sequences/). Stem-loop reverse transcription (RT) primers were designed according to Chen [19]. The RT reaction conditions used involved incubations at 16°C for 30 min; 37°C for 30 min and then 72°C for 10 min. The thermal cycling protocol for the PCR involved an initial denaturation step at 95°C for 5 min followed by 40 cycles at 95°C for 30 s, 57°C for 30 s and 72°C for 30 s. The melt curves for each PCR were carefully analyzed to determine any non-specific amplification. The expression of each miRNA was calculated using the 2^−ΔΔ*C*^_T_ formula and normalized to U6 snRNA expression [20].

**Figure 4:**
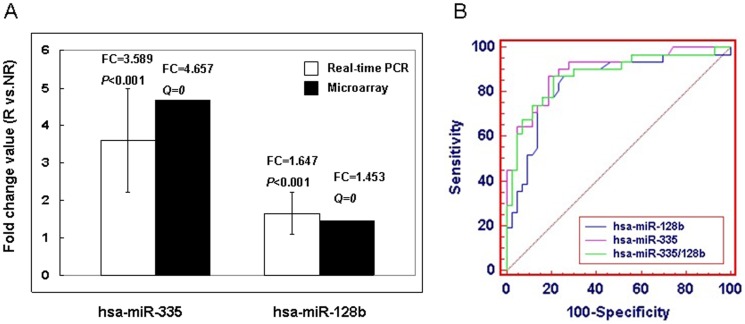
Comparison of FC value and ROC curves of hsa-miR-335 and hsa-miR-128b. **A.** The FC value of hsa-miR-128b and hsa-miR-335 were 1.647 and 3.589 when compared recurrence with non-recurrence samples based on real-time PCR data, respectively. Consistent results were observed on microarray data. **B.** hsa-miR-335 was more sensitive and specific than hsa-miR-128b and hsa-miR-335/128b in classifying recurrence and non-recurrence samples.

### Unsupervised Algorithm

SAM [21] was used to perform the unsupervised clustering calculation. The cutoff for significance was determined by a tuning parameter delta, chosen by the user based on the false positive rate and represented by the q value. We chose a fold change greater than 2, and q = 0 as the selection criteria for the differential expression of miRNAs.

**Figure 5:**
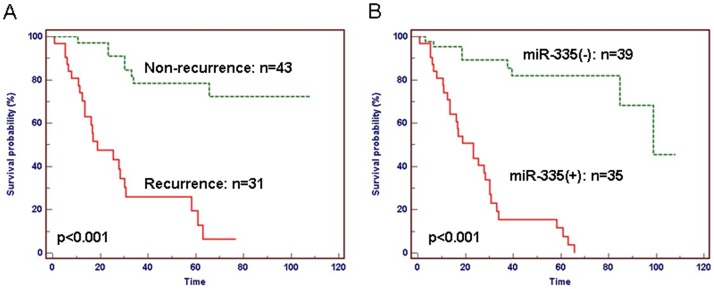
Kaplan-Meier survival curves of GC patients correlated with the clinical outcome and expression levels of hsa-miR-335. A. Survival curve of recurrence group compared with that of non-recurrence group in GC patients. A significant difference of survival time was observed between GC patients with and without recurrence (*P*<0.001). B. Survival curve of miR-375(+) group compared with miR-375(−) group in GC patients. miR-375(+) group has poor prognosis compared with miR-375(−) group (*P*<0.001).

### ROC and Kaplan-Meier Survival Curve Analysis

ROC curve analysis was conducted using the MedCalc software packages (verison 8.2.1.0; Mariakerke, Belgium). The AUC curves provided a measure of the overall performance of a diagnostic test. The ratio of miRNA signal intensities and Ct value of each miRNA were used for ROC calculation in samples. Survival analyses were also performed by the MedCalc software package.

### Statistical Analysis

The clinical data were analyzed using the Chi-Square test. The cumulative survival curve was compared by the log-rank test. For all analyses, a difference with *P*<0.05 was considered statistically significant.

**Figure 6:**
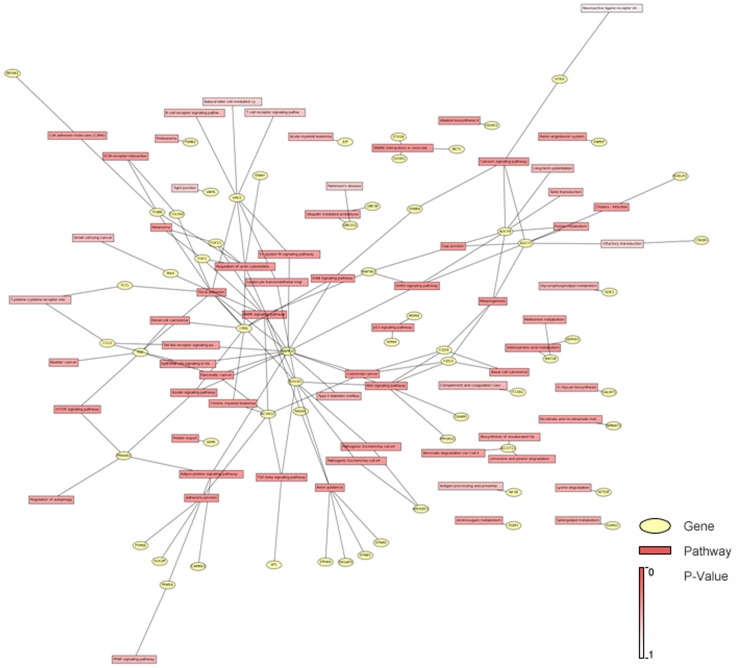
Molecular signal-pathway networks regulated by hsa-miR-335. The main framework of this pathway network include of p53,MAPK, TGF-β, Wnt, ERbB, mTOR, Toll-like receptor, focal adhesion.

**Table 4:** Signaling pathway analyses of hsa-miR-335 targeted genes.

Pathway	Gene	p-Value	q-Value
Focal adhesion	FLT1;MAPK10;ROCK1;COL5A2	1.21E-06	4.58E-05
	PGF;CRKL;VAV2;ITGB8		
MAPK signaling pathway	MAX;RASA1;MAPK10;MAP3K2	1.05E-05	8.14E-05
	CRKL;ACVR1C;FGF2;FGF2		
Regulation of actin cytoskeleton	ROCK1;CRKL;FGF2;FGF2	2.02E-05	1.34E-04
	ENAH;VAV2;ITGB8		
Wnt signaling pathway	DAAM1;FZD3;FZD3;MAPK10	2.53E-05	1.47E-04
	ROCK1;PPP2R5C		
TGF-beta signaling pathway	SP1;ROCK1;ACVR1C	0.00438	0.00506
ErbB signaling pathway	MAPK10;ERBB4;CRKL	0.00438	0.00506
Calcium signaling pathway	ADCY3;ADCY3;ERBB4;HTR4	0.00525	0.00569
mTOR signaling pathway	PRKAA2;PGF	0.01644	0.01233
Basal cell carcinoma	FZD3;FZD3	0.01827	0.01323
p53 signaling pathway	MDM4;RPRM	0.02790	0.01753
Epithelial cell signaling in H pylori infection	MAPK10;CCL5	0.02865	0.01788
Toll-like receptor signaling pathway	MAPK10;CCL5	0.05657	0.02712
Cell adhesion molecules (CAMs)	NRXN1;ITGB8	0.09066	0.03470
B cell receptor signaling pathway	VAV2	0.24601	0.07614
T cell receptor signaling pathway	VAV2	0.33671	0.09647
Natural killer cell mediated cytotoxicity	VAV2	0.40544	0.10743

**Table 5:** Gene ontology analyses of hsa-miR-335 targeted genes.

GO Term	Input Symbol	p-Value	q-Value
GO:0007155 cell adhesion	PCDH9;NRXN1;NRXN1;CCL5	2.26E-11	5.40E-11
	LSAMP;EFNB1;FAT3;PCDH19		
	PCDH19;SSX2IP;ITGB8		
GO:0007049 cell cycle	CHFR;CCNF;TFDP2;EVI5;	1.45E-09	2.94E-09
	AIM1;ARHGEF2;STRN3;FOXN3		
	SETD8;CCNT2		
GO:0030154 cell differentiation	FLT1;KAZALD1;SPATA2;ZNF3	7.66E-08	1.19E-07
	DCX;PGF;ACVR1C;PPP1R9B		
	EFNB1;EFNA5		
GO:0007267 cell-cell signaling	CCL5;ENPEP;PGF;EFNB1	4.70E-07	6.43E-07
	EFNA5;FGF2;PGR		
GO:0051301 cell division	CHFR;CCNF;EVI5;ARHGEF2	4.78E-08	8.01E-08
	SETD8;CCNT2		
GO:0008283 cell proliferation	FZD3;EVI5;ENPEP;ERBB4	5.79E-05	4.16E-05
	PRKD1;EIF5A2		
GO:0006915 apoptosis	MDM4;ROCK1;TIA1;PDCD7	5.18E-04	2.73E-04
GO:0016055 Wnt receptor signaling pathway	CPZ;FZD3;FZD3;PYGO2	3.54E-06	3.96E-06
GO:0001525 angiogenesis	HAND1;ENPEP;PGF	2.35E-04	1.36E-04
GO:0000187 activation of MAPK activity	FRS2;FGF2;CAMKK2	0.001195	5.01E-04
GO:0007254 JNK cascade	MAPK10;CRKL	0.001230	5.12E-04
GO:0007050 cell cycle arrest	RPRM;PPP1R9B	0.002458	7.59E-04
GO:0001558 regulation of cell growth	KAZALD1;CRIM1	0.004463	0.001049
GO:0001568 blood vessel development	GJA5;CRKL	0.011243	0.002030
GO:0006281 DNA repair	FANCA;SMG1	0.016454	0.002585
GO:0016477 cell migration	FLT1;ENPEP	0.019502	0.002910

### miRNA Targeted Gene Prediction and Signal Pathway Analyses

We utilized a miRNA target gene prediction database TargetScan (http://www.targetscan.org) to select plausible targets of the differential expressed miRNAs. An integrated gene ontology database molecular annotation system (MAS3.0, http://www.capitalbio.com) was used to investigate the miRNA targeted genes and their involvement in various signal pathways.

## Results

### Clinical Characteristics of GC Patients

A total of 74 patients with/without recurrence were selected for systematic analysis. 31 patients had recurring GC that was proven pathologically by biopsy at anastomosis sites via endoscopy. 43 patients without recurrence were selected as the control group with matches in sex, age at diagnosis, TNM staging, treatment and the number of involved lymph node (Table 1).

There were no significant difference in the Sex (*P* = 0.469), Age (*P* = 0.502), Tumor location (*P* = 0.299), Differentiation (*P* = 0.511), Lymph node resection (*P* = 0.217) and Status of adjuvant chemotherapy (*P* = 0.214). There were significant difference in UICC stage (*P* = 0.108) and survival/death ratio noted (8/23 in recurrence group vs. 34/9 in non-recurrence group, *P*<0.001), with median survival time of 18.9 months in recurrence vs. 43.9 months in non-recurrence group respectively (*P*<0.001) (Table 1).

### miRNA Expression Profiles Associated with GC Recurrence

We used a miRNAs microarray chip to measure miRNAs expression levels in 10 GC samples with/without recurrence (5/5) as training set. When comparing miRNA expression between the groups, 7 up-regulated and 5 down-regulated miRNAs were found that were statistically significant in the GC recurrent group (Table 2). These 12 miRNAs could be used to discriminate between GC with and without recurrence based on a hierarchical cluster analysis (Figure 1).

### hsa-miR-335 is the Best Signature for Classifying GC with and without Recurrence in Training Set

We used ROC method to analyze the sensitivity and specificity of the candidate biomarkers based on miRNA microarray data. We obtained 2 miRNAs hsa-miR-335 and hsa-miR-128b with best sensitivity and specificity in classifying GC samples with and without recurrence (Figure 2, Table 3). Combined with the FC value (FC_ hsa-miR-335_ = 4.675; FC_ hsa-miR-128b_ = 1.453) of each biomarker, we select hsa-miR-335 for further study.

### Validation of the Signature in Test Samples by Real-time PCR

The expression level of hsa-miR-335 was detected by real-time PCR in 64 test GC samples compared with the matched adjacent tissue as control. The results showed that in 26 cases with recurrence, 21 cases (21/26, 80.8%) were high-expressed of hsa-miR-335; and in 38 cases without recurrence, 29 cases (29/38, 76.3%) were low-expressed of hsa-miR-335 (Figure 3). The same results were observed in blood samples using a mixed-blood sample from 20 non-cancer patients with digestive diseases as control: In 26 cases with recurrence, 19 cases (19/26, 73.1%) were high-expressed of hsa-miR-335; and in 38 cases without recurrence, 30 cases (30/38, 78.9%) were low-expressed of hsa-miR-335 (Figure 3). There were 11 cases (11/64, 17.2%) not matched in the expression level of hsa-miR-335 in comparison of the results between frozen fresh and blood samples.

In addition, we detected the expression level of hsa-miR-128b in 64 GC blood samples using real-time PCR. The results showed that in 26 cases with recurrence, 18 cases (18/26, 69.2%) were high-expressed of hsa-miR-128b; and in 38 cases without recurrence, 27 cases (27/38, 71.1%) were low-expressed of hsa-miR-128b. The FC value of hsa-miR-128b and hsa-miR-335 were 1.647 and 3.589 when compared recurrence with non-recurrence samples, respectively (Figure 4A). We also analyzed the sensitivity and specificity of hsa-miR-335, hsa-miR-128b and hsa-miR-335/128b (combined with miR-335 and miR-128b as a signature) based on the real-time PCR data. The results showed that hsa-miR-335 was more sensitive and specific with an AUC value of 0.88 than hsa-miR-128b (AUC = 0.79) and hsa-miR-335/128b (AUC = 0.84) in classifying recurrence and non-recurrence samples (Figure 4B, Table 3).

### hsa-miR-335 as a Disease Progression Signature to Predict Recurrence Risk in GC Patients

The 74 GC patients were divided into two groups, including 31 patients with recurrence as a group and 43 patients without recurrence as a group. The patients who experienced a recurrence of GC had a significantly reduced median survival rate (*P*<0.001; Figure 5A). We detected the expression levels of hsa-miR-335 in all 74 GC samples. A significant difference has observed in two groups which represent high-expressed of hsa-miR-335 group and low-expressed of hsa-miR-335 group as follows: miR-335(+) and miR-375(−). GC patients (n = 35) with miR-335(+) had significantly reduced median survival compared to those (n = 39) with miR-375(−) (*P*<0.001; Figure 5B).

### Signaling Pathway and Gene Ontology Analyses of hsa-miR-335 Targeted Genes

In order to investigate the possible regulation mechanisms of the hsa-miR-335 in the process of GC recurrence, we utilized a bioinformatics database to select plausible targets of this miRNA. A total of 255 genes were predicted as the target genes of hsa-miR-335. Signaling pathway analyses showed that most of the targeted genes which were regulated by hsa-miR-335 were involved in the same pathways, such as p53,MAPK, TGF-β, Wnt, ERbB, mTOR, Toll-like receptor, focal adhesion (Table 4, Figure 6). We proposed that these multiple pathway alterations might be involved in clinical pathological features and patients’ outcome of GC. Meanwhile, we used an integrated gene ontology database to annotate the molecular function of the miRNA targeted genes. The results showed that genes regulated by hsa-miR-335 participated in most of the important biological process associated with human cancer (Table 5).

## Discussion

Recurrence is frequent in GC patients following surgery; therefore it is important to identify cases with a high recurrence risk. Traditional clinic pathological factors are sometimes inadequate for prediction of recurrence in individuals and many research groups have attempted to identify biomarkers using new technologies that might distinguish these high-risk cases. A number of recent investigations have documented miRNA alterations that are involved in the initiation and progression of human cancers. miRNA expression profiling of human tumors have identified expression signatures associated with diagnosis, staging, progression, prognosis and response to treatment.

In this study, we analyzed primary GC cases in order to predict disease recurrence and defined non-recurrent cases as those free of GC for at least one year after curative resection. We identified hsa-miR-335 as a plausible prognosis signature of GC based on the microarray data from 10 GC samples as training set. Furthermore, we validated this signature on 64 test samples with more than an average of 85% sensitivity and specificity. The survival analyses results showed that patients with high expression levels of hsa-miR-335 had reduced overall survival rate compared to those with low expression levels of hsa-miR-335. These results indicate that this signature had the potential to predict recurrence of GC.

Microarray technology has developed significantly and become a comprehensive and useful method to help us better understands cancers [22]. It is now widely used to study the functional roles of miRNAs in many types of cancer [23], with several significant miRNA effects on oncogenes and tumor suppressor genes identified. Of the 12 miRNAs identified in this study, hsa-miR-373 was one of the down-regulated miRNAs in the recurrent group. This may be an oncogene functioning through the well-known p53 pathway that is involved in carcinogenesis [24]. Some miRNAs, such as hsa-miR-19a and hsa-miR-32, are located in cancer-associated genomic regions, which might be involved in malignancies via deletion, amplification, or epigenetic modification mechanisms [25]. Additionally, hsa-miR-429 were up-regulated in recurrent cases and clustered on chromosome 1, confirming that some miRNAs such as hsa-miR-200b are clustered with hsa-miR-429 and able to co-regulate their targeted genes [26].

Until now, the miRNA designated in this study to predict the risk of GC recurrence have not been well-characterized. hsa-miR-335, which is transcribed from the genomic region chromosome 7q32.2, has been reported to differential expressed in benign and malignant tumours [27]. Importantly, it is also demonstrated that miR-335 regulates Rb1 and controls cell proliferation in a p53-dependent manner [28]; participates in the development of breast cancer [29]; regulates growth and invasion of malignant cells [30]. The latest research reports that miR-335 might function as a metastasis suppressor in GC by targeting Bcl-2 and SP1, and could be further developed as a potential prognostic factor [31]. To summarize the views above, the function of miR-335 was controversial. Our results shows that hsa-miR-335 was up-regulated in GC recurrence samples and involved in most of the oncogenic signaling pathways, such as p53, MAPK, TGF-β, Wnt, ERbB, mTOR, Toll-like receptor, focal adhesion. These multiple signal pathway alterations, especially including p53 pathway, might reasonably affect clinical outcome including GC recurrence risk [32]. Although the prognostic role of hsa-miR-335 in gastric cancer is unknown, our findings are encouraging.

Blood is the most accessible sample in hospitals. The clinical significance of identification of biomarkers which could be easy detected just as hsa-miR-335 is obviously. In addition, archived paraffin-embedded specimens are also one of the readily available materials in hospitals, which are often kept with well documented clinicopathological data. They represent excellent sources for prognostic application in both basic research and clinical studies. miRNAs range in size from 19–25 nt and they are protected by the RNA-induced silencing complex, which may render them less susceptible to RNA degradation compared to mRNA in these tissues [33]. Thus, we were able to detect miRNA expression in paraffin-embedded samples and focused on the systematic analysis using this signature for relevant prognostic value. The PCR-based classification method therefore, appears to provide us with a way, using easily available clinical specimens, to predict disease recurrence.

In summary, we have identified a prognosis-related miRNA which can predict recurrence risk of GC patients. By combining this signature with conventional clinicopathologic factors, we should be able to predict a patient’s disease outcome more accurately. Additionally the identification of high-risk patients would lead to the consideration of additional therapeutic intervention and may thus inform the physician of a better follow-up program.
